# What Can the Legisiature Do for Us?

**Published:** 1834-07-01

**Authors:** 


### MEDICAL POLITICS AND INTELLIGENCE.

### WHAT CAN THE LEGISLATURE DO FOR US ?

We fear that if an impartial estimate were made of all that can be done for the medical profession by force of law, the catalogue of possibilities would bear too great a resemblance to Martial's review of his own works:

Sunt bona, sunt quædam mediocria, sunt mala plura.

In the first place, the *bona* are, the repeal of the Apothecaries' Act, that epitome of mischievous absurdities. By repealing this monstrosity, we get rid of the apprentice-system, and youths of sixteen, destined for the practice of physic, will no longer spend five years in mixing medicines, as this is an art that may be learned admirably well in three months.

Then for the *mediocria*, we may have some little alterations in names and titles; a wholesale M.D. manufactory will perhaps be set up in London, and diplomas may be granted on terms that will utterly ruin the established factories in the North. This will make a number of people very happy for six months, till they wake from their dream of dignity, and regret the few but solid sovereigns which have left an empty parchment as their substitute.

And now, alas! for the *mala plura.* We fancy that a crusade against unlicensed practitioners would be very agreeable to many of the so-called medical reformers; for, as the physicians, a century and a half ago, found themselves undersold by the apothecaries, and attempted, but in vain, to check the plebeian innovation by verdicts and decisions, so now the apothecaries complain that the druggists are ousting them from practice, and that a man who knows no more than an apothecary of Queen Anne's days, will make his comfortable £1000. per annum by counter-practice, with occasionally a few snug visits under the rose. According to Gray, indeed, in the preface to his Supplement to the Pharmacopœia, the druggists' practice is legal, as the Apothecaries' Act was restricted to those who "practise as apothecaries," and "à fortiori, the midwives, herbalists, cuppers, barbers, electricians, galvanisers, dentists, farriers, veterinary surgeons, village wisemen, and cow-leeches, are left in full possession of their ancient practice, and may be employed by those who place confidence in them, as they cannot be confounded with apothecaries, though the chemist and druggist may." (Fourth edit. p. xxvii.)

We would not, however, recommend any of the village wisemen, or other of the younger sons of Galen included in the above quotation, to risk a visit to the Court of King's Bench on Mr. Gray's authority; for, though his treatise is excellent in pharmacology, it is by no means a text-book in law.

Still, legally or illegally, the druggists must and will practise, and, we will add, ought to practise; as long as extreme cheapness is essential to many customers, so long must slight or coarse goods be got up for their use, all Acts of Parliament to the contrary notwithstanding. Everybody would like to have the advice of the King's physician rather than go to Mr. Bolus, but Mr. Bolus gives credit, and this is a *sine quâ non* with many patients. Other sick again have neither a guinea for the first, nor courage to face the voluminous bill of the second; but they have ninepence, and this will buy them the somewhat hasty, yet perhaps decent empirical advice of a druggist, gabbled across his counter, and terminating in the exhibition of a vigorous black draught.

The cant of pretended reformers about "protecting the public from irregular practitioners," is evidently based on the false supposition that patients are quite in the dark as to the relative merits of medical men; but this is rarely the case. A man afflicted with cynanche, though his capacity were as straightened as his fauces, would willingly seek the advice of a Baillie or an Armstrong; it is the *res augusta domi* which compels him to go to somebody in a court, or to buy a box of black currant lozenges, or to let his throat get well of itself.

We should be glad to know, too, by what unheard-of severities, by what Russian discipline, it is proposed to put down all practice, save by men with properly sealed parchments? Are we to have officers of medical excise, empowered to enter houses strongly suspected of harbouring unlicensed medicine-chests? Or will my Lady Bountiful be allowed (like the master of a house under the Conventicle Act,) to physic her household and sundry poor neighbours, provided that her hospital does not exceed twenty beds? Shall we hear of the triumphant seizure of a cask of pennyroyal water, or will rhubarb pies be searched, charged on oath with being made with the root instead of the stalk?

In Spain, indeed, a dose of tartar emetic cannot be got without a physician's prescription; and a learned friend of ours, who found himself compelled to put some of that triple salt into his wine, as a thief-trap, was obliged to lay a deep scheme to obtain possession of it. It is impossible to imagine that the hideous chimeras of such *soi-disant* medical reformers can be realised; yet we fear that our profession, in calling for the aid and protection of the legislature, will find themselves in the condition which history and fable alike point out as the inevitable destiny of the weak, who intrust the management of their affairs to the strong.

